# The influence of a serious game’s narrative on students’ attitudes and learning experiences regarding delirium: an interview study

**DOI:** 10.1186/s12909-020-02210-5

**Published:** 2020-09-01

**Authors:** Kiki R. Buijs-Spanjers, Anne Harmsen, Harianne H. Hegge, Jorinde E. Spook, Sophia E. de Rooij, Debbie A. D. C. Jaarsma

**Affiliations:** 1 Department of Geriatric Medicine, University of Groningen, University Medical Center Groningen, PO Box 30001, HPC: AA43, Groningen, 9700 RB The Netherlands; 2 Center for Education Development and Research in Health Professions, LEARN, University of Groningen, University Medical Center Groningen, Groningen, The Netherlands; 3 Strategic Communication Group, Wageningen University, Wageningen, The Netherlands; 4 Medical School Twente, Medical Spectrum Twente, Enschede, The Netherlands

**Keywords:** Delirium, Education, Serious game, Narrative

## Abstract

**Background:**

Delirium is a neuropsychiatric syndrome that affects patients’ attention and awareness as a result of a physical condition. In recent years, persistent gaps in delirium education have led to suboptimal delirium care. Still, little is known about what are the most important aspects of effective delirium education. Serious games are both entertainment and an interactive, safe learning environment where players can experiment and create new knowledge. They have the potential to contribute to improved delirium education. We used a video-based serious games’ narrative to explore aspects essential to enhance students’ attitudes and learning experiences regarding delirium.

**Methods:**

We created a semi-structured interview guide and interviewed seven nursing and nine medical students about their attitudes and learning experiences, after they had played the game. A qualitative descriptive design and inductive content analysis with constant comparison were used.

**Results:**

The patient’s and nurse’s perspective, interactivity to experiment, realistic views on care options, and feedback on care actions were important for enhancing students’ attitudes and learning experiences regarding delirium. Students felt these aspects encouraged them to get actively involved in and experiment with the study material, which in turn led to enhanced reflection on delirium care and education. Our findings highlight the importance of a more patient-oriented focus to delirium education to drive attitudinal change. Students’ learning experiences were further enhanced through their affective responses provoked by the perspectives, interactivity, realism, and feedback.

**Conclusions:**

Students considered the characters’ perspectives, interactivity, realism, and feedback important aspects of the game to enhance their attitudes towards delirious patients and enrich their learning experiences. A patient-oriented narrative provides a clinically relevant experience in which reflection plays an important role. The serious game also serves as medium to actively experiment with care solutions to create better understanding of how healthcare professionals can influence a delirious patient’s experience.

## Background

### Delirium

In recent years, researchers have identified persistent gaps in delirium education for healthcare professionals, which has led to suboptimal delirium care [1–7]. Delirium is an acute neuropsychiatric syndrome characterized by altered attention, awareness, and cognition. It is associated with serious consequences such as increased length of hospital stay, functional decline, and mortality [8]. Delirium has three subtypes: hyperactive, hypoactive, and mixed delirium [9]. Patients with hyperactive delirium are restless, agitated, and hyper alert. Patients with hypoactive delirium seem to be lethargic and drowsy, and sometimes they appear to be sedated. Patients with mixed delirium show hyperactive as well as hypoactive symptoms [9]. Depending on the clinical setting, the incidence of delirium varies from 29 to 64% in medical and geriatric wards, up to 80% in Intensive Care Units. It is also present in about 40% of nursing home residents [10]. Experiencing delirium and having flashbacks of delirious episodes is frightening for patients and affects their psychological and emotional well-being [11–13]. Providing appropriate care to delirious patients imposes a great burden on healthcare professionals, for example, because of difficult behaviors of patients and overlapping symptoms with dementia [14, 15].

Although early detection of delirium or timely recognition of patients at increased risk of delirium is highly important to prevent incidence or severity of delirium, it frequently goes unrecognized [16]. Low detection rates of delirium are often related to low delirium awareness, inadequate knowledge and education, lack of time, feeling uncomfortable with delirium assessment, and missing co-operation between professions. However, if the presence of delirium and/or increased risks are detected, such barriers – together with negative attitudes towards delirium – are also related to suboptimal delirium management [2, 14, 17–19].

Researchers emphasize the need for different and more effective education to overcome unnecessary barriers to delirium detection and management [4, 6]. On the one hand, the content of delirium education needs to be modified. It is important to pay more attention to negative attitudes of healthcare professionals towards delirium, the lack of understanding of how frightening delirium is, and behavior and communication skills in delirium care [4, 6]. On the other hand, there is a call for educating delirium care using methods that incorporate interaction and active involvement of learners, sufficient feedback loops, and simulations [3, 6, 14, 20, 21]. Delirium education should become more patient-oriented instead of only focusing on specific disease-related knowledge [4]. To that aim, it is important to involve patients and family members in the delivery and development of delirium education and increase clinical experiences [3, 6, 22]. However, this would increase the burden on patients [3, 22]. Serious games provide a safe environment where players can practice and gain experiences without increasing the burden on patients [23].

### Serious games

Serious games are intended to provide playful learning experiences. They are not only learning environments, but also interactive applications with a characterizing goal other than pure entertainment [23, 24]. The goal can vary from improving physical health to learning or changing behavior. In medical education, serious games are often more effective in achieving educational goals than conventional methods [25–27]. In previous research, we showed that the serious game ‘The Delirium Experience’ is appropriate for teaching delirium care [28]. Still, little is known about which aspects enhance delirium care [1, 3, 4], and about which aspects of serious games contribute to players’ learning experiences and outcomes [29–31]. Promising aspects of serious games for instructional techniques to enhance learning and motivation are, for example, assessment, collaboration, feedback, level of realism, reflection and narrative [32]. In this study, we will mainly focus on the narrative. The narrative is a game element that can be of value in addressing the issues in delirium education. It supports players in processing information and making sense of their experiences [33, 34]. Besides, experiential learning seems to be important. Healthcare professionals with insufficient knowledge or experience often rely on experiential learning when they provide care to delirious patients [14, 35, 36].

#### Narratives

Narratives can be defined as stories with a beginning, middle and end. They provide information on scenes, characters, and conflict [37]. In addition, they raise questions or unresolved conflicts and present a solution [37]. They often increase involvement with the learning material and create empathy for the characters through both narrative characteristics (i.e., characters and interactivity) and engaging elements of the narrative (i.e., identification, transportation, and perceived realism) [38–41]. In medical education, narratives may be particularly relevant in promoting the more humanistic aspects of medicine [42].

When learners get immersed into the narrative, it can influence their attitudes [37]. Attitudes are learned evaluations of persons, places or issues that affect thoughts and behaviour [43]. They are composed of affective (feelings and emotions), behavioural and cognitive components (knowledge and beliefs) [44, 45]. A narrative about patients, families, and carers involved in delirium care may stimulate attitudinal change by letting players learn from the patient’s experience, which is essential for improving delirium care [6, 46]. A first-person perspective in a narrative facilitates better comprehension of the study material by enabling transfer of what players have seen towards their own performance [47]. A first-person perspective of people involved in delirium care, therefore, holds the potential to enhance players’ understanding of the patient’s experience and behaviour, and communication skills in delirium care.

Although the use of narratives in education and serious games seems promising, researchers argue that it also can result in the player losing the focus on learning experiences and outcomes [38]. Hence, more research is needed to understand how these narratives influence learning experiences and outcomes, and what aspects play an important role [32, 38].

#### Experiential learning

Learning within a serious game can often be considered experiential learning. Kolb’s theory of experiential learning explains how learners take experiences they have had in real life into their learning process [48]. Experiential learning is an active form of learning, in which learners’ experiences must be interpreted and integrated with what they already know, to create new knowledge. Reflection is essential to make sense of experiences [48, 49]. Another important aspect of experiential learning is that learners have opportunities for experimentation with new ideas and concepts that are formed through their experiences and interpretations of these experiences [48].

This type of learning is used in many fields of healthcare professions education [49, 50]. Healthcare professionals describe that they often rely on experiential learning when they provide delirium care, especially in cases where they lack education or experience to provide good quality care [14, 35, 51]. However, experiential learning is not often explicitly incorporated into delirium education [14, 36, 51].

### Aim

In this study, we aimed to explore aspects that could potentially play a role in enhancing students’ attitudes and learning experiences regarding delirium by making use of a serious game’s narrative.

## Methods

### Study design

In this study, we explored which aspects of the serious game’s narrative influenced students’ attitudes and learning experiences regarding delirium. Therefore, we needed to engage in interpretations of participants’ experiences after playing the serious game. To do so, a qualitative descriptive method was used [52]. We conducted semi-structured interviews, as these allowed us to engage in participants’ interpretations, probe for responses and clarify ambiguities [53, 54].

### Game and narrative description

The Delirium Experience is a serious game that makes use of video simulation. The main aims of the game are to increase knowledge and drive attitudinal change by increasing players’ understanding of the patient’s experience. It is intended to teach players how to improve their care for delirious patients and to provide insight into both what patients endure during delirious episodes and how healthcare professionals’ actions influence the patient’s experience [55]. The game adheres to the delirium guidelines of the United Kingdom [56], and the Netherlands [57]. The scenes are based on experiences of real patients who experienced delirious episodes. The trailer can be found at: https://www.youtube.com/watch?v=A-lLLP8Me0E.

The game includes the narratives of an older patient undergoing a hip surgery and a healthcare professional who has to provide care to this patient. After the surgery, the patient experiences delirious episodes, showing the mixed subtype of delirium. The episodes differ in severity, depending on the care provided. The game represents four working days of a healthcare professional. From this perspective, players have to provide care to the patient and all actions they take will manipulate the narrative. During the corresponding nights, players receive the patient’s perspective. The features can differ, depending on the player’s care actions during daytime. After each play day of the game, players receive tailored feedback on how their care can be improved. In addition, players receive feedback through the patient’s responses to the their actions and the impact of their actions on the delirium symptoms. At the end of the game, final written feedback on all chosen care options is provided within the game. It takes 20 min to complete the game once.

### Participants

The Delirium Experience is intended for a broad audience, varying in background, experience and education. Researchers have emphasized the need for educating delirium care to healthcare professionals in an early stage [6]. We were therefore particularly interested in the influence of the narrative on Vocational Nursing and Master of Medical Sciences students. In the Netherlands, nursing education is offered in two ways: Vocational Nursing education and the Bachelor of Science in Nursing. The focus of Vocational Nursing education is more on applying practical knowledge, whereas the focus of the Bachelor of Science in Nursing is more on applying scientific knowledge to practice. With these two target groups, we were able to gather a heterogenic sample of Dutch students at either a Vocational Nursing school or a University Medical Center.

In total, 16 students participated in our study. The sample consisted of seven nursing and nine medical students, of which two were males and 14 females in the age of 16–27 years. This reflects the gender distribution of students in nursing and medicine in the Netherlands. Table 1 shows an overview of the participants’ characteristics.

**Table 1:** Characteristics of participants and interviews

Participant	Education type	Years of education	Experience with delirium	Age (in years)	Interview duration (in minutes)
P1	Nursing	1	Some	17	16
P2	Nursing	1	Some	17	13
P3	Nursing	1	No	18	21
P4	Nursing	1	No	18	26
P5	Nursing	1	No	16	26
P6	Nursing	1	No	20	35
P7	Nursing	1	No	17	22
P8	Medical	4	Yes	22	32
P9	Medical	4	Some	22	25
P10	Medical	4	Yes	23	23
P11	Medical	4	Some	23	16
P12	Medical	6	Yes	25	40
P13	Medical	4	No	23	41
P14	Medical	6	Yes	24	37
P15	Medical	5	Yes	23	46
P16	Medical	6	Some	27	36

### Participant recruitment and setting

Purposive and convenience sampling was used to recruit participants varying in background, experience, and education [53]. For the purposive sample, we used practical sessions that were part of the regular delirium education, where students could play the game once or twice. Furthermore, we used convenience sampling to reach medical students via contacts at our Geriatric Department.

The practical on delirium for nursing students took place in June 2018, for medical students in July 2018. A week before the practical, students were informed about the study and received an e-mail with an information letter. If they needed additional information or were willing to participate in the study, they could e-mail the second author (AH). At the beginning of the practical, the study was explained once more. Out of 40 students participating in these practicals, 11 were willing to participate. Although students were not asked to provide a reason for non-participation, some students voluntary stated that they did not want to participate due to time-constraints.

From the interviews with the initial 11 students, new themes emerged. In January and February 2019, we therefore reached out to additional medical students in the network within our Geriatric Department. Students who were interested in participating received an e-mail with an information letter and could contact the first author (KBS) for additional information or to schedule an appointment for the interview. We included five additional medical students until data saturation was reached and no new themes emerged from the interviews. The interviews took between 13 and 46 min.

### Ethical considerations

Ethical approval or registration was not necessary in accordance with the International Committee of Medical Journal Editors (ICMJE) guidelines as our participants were medical and nursing students who voluntarily chose to participate.

To ensure students did not feel obliged to participate, all students were allowed to play The Delirium Experience without participating in the study. Furthermore, the researchers were neither involved in the participants’ education nor did they know them beforehand. All participants signed an informed consent form stating that they were willing to participate and allowed audio-recordings of the interview. Participants were guaranteed that all data would be processed anonymously and confidentially, and that participation was voluntary. They could withdraw from the study at any time, without having to provide a reason.

### Research team and reflexivity

The team was composed of two junior (KBS and AH) and four senior researchers (HH, JS, SR, DJ). KBS has a background in communication sciences and nutrition, and she was a PhD-candidate at the time of the interviews. AH is a nurse and has a background in communication sciences, and she was a Master of Science student at the time of the interviews. HH is an internist and clinical educator. JS is an experienced researcher in serious game research. SR is a professor of medicine, trained in geriatrics and internal medicine. And DJ is a professor in health professions education. The diversity of this research team enabled us to interpret the results with insights from the different aspects of this narrative: medical, educational, and as a serious game.

To prevent recall bias, interviews were conducted within 1 week after playing the game. Furthermore, interviews were conducted at quiet locations that suited the participant without the presence of other people to make sure participants felt at ease and not distracted during the interview. We also explained to participants that the researchers conducting the interviews (KBS and AH) were not involved in the development of The Delirium Experience to prevent interviewer bias leading to socially desired answers.

### Data collection

The first and second author conducted the interviews, AH conducted nine and KBS the remaining seven. To ensure consistency across interviews, we used a semi-structured interview guide (Additional file 1), based on literature and discussions among team members (KBS, AH, JS, DJ). This guide specifically included questions on participants’ attitudes and behaviors towards delirious patients, and their knowledge on and learning experiences with delirium [4]. We used narrative characteristics (i.e., characters) and engaging elements of a narrative (i.e., realism) as prompts to provoke more in-depth answers [39, 40]. We conducted two pilot interviews to refine the interview guide. Before the interviews started, participants answered demographic questions on age, gender, education level and year, and experience with providing care to delirious patients. All interviews were audio-recorded, transcribed verbatim, and summarized.

### Data analysis

Data collection and analysis occurred concurrently. To analyze the data, we used thematic inductive content analysis with constant comparison to find similarities or differences within and across interviews [53, 58]. First, two researchers (KBS and AH) thoroughly read all the transcripts to get familiar with the data.

Thereafter, codes were assigned to basic aspects of the content. The second author (AH) initially coded the interviews. Due to time constraints, she was not able to code all 16 transcripts, but coded eight. To ensure consistency, she and the first author (KBS) coded three transcripts together and then the first author coded the remaining five transcripts. The codes were repeatedly evaluated by four researchers (KBS, AH, JS, DJ). Disagreements on assigned codes were solved through discussion. New codes that emerged from the data were applied inductively. We used Atlas.ti software, version 8 (ATLAS.ti Scientific Software Development GmbH, Berlin) to facilitate the coding process.

Subsequently, overarching themes were defined and named. Table 2 shows the themes and their corresponding codes. Team meetings were used to discuss the process and interpretations of the data. Summaries of all transcripts were discussed by two researchers (KBS and HH) to clarify the themes.

**Table 2:** Themes and codes used in data analysis

Theme	Code
Attitudes	Affective
	Behavioral
	Cognitive
Narrative characteristics	Characters
	Interactivity
Engaging elements	Identification
	Transportation
Learning experience	Concrete experimentation
	Reflective observation
	Abstract conceptualization
	Active experimentation

## Results

We were able to distinguish four aspects affecting students’ attitudes and learning experiences regarding delirium: (1) perspectives of the characters, (2) interactivity to experiment, (3) realism, and (4) feedback.

### Perspectives of the characters

Within the game, players received the perspective of two characters, the patient and the nurse. Both perspectives were often mentioned. They made participants more involved in the narrative. Being able to see the same scene from two different perspectives was highly valued by the participants, because the patient’s responses made more sense to them now. As explained by a participant: *“First, you just see the teabags from his [the patient] perspective. Then from the nurse’s perspective, you see him muddle with his hands … you think the patient is just acting weird, but when you also see what it’s like for the patient, it makes sense that you tidy up if you see teabags.” [P12]* Participants also stated that the two perspectives made them aware of the consequences of their actions as a healthcare professional, as one participant explains: *“When I played the second time, I played dark play, and I thought ‘if you act incorrectly, the things people see are really more frightening’. This made me realize that in a certain way you are responsible for how intense delirious episodes become.” [P11]*

#### Patient

The patient’s perspective was highly valued by participants. It enabled them to create an idea of what delirious patients see and endure when experiencing delirious episodes. A participant explains this by comparing it to reading learning material: *“If you’re reading, you have to imagine what it’s like … and in ‘The Delirium Experience’, you can see what a patient sees, how it looks like and what happens … it makes it easier to create an image of what it would be like for a patient.” [P13]* Because of this, participants felt more empathy for delirious patients. It also made them realize that experiencing delirious episodes is more intense and frightening than they expected. Explained by two participants: *“What we learned was that, just in general, a delirious patient can be a bit confused, but it’s more than confusion, for the patient it’s really intense.” [P5]* and *“When you leave during the night, you don’t see delirium anymore, but for the patient it’s most intense at that time. That’s something I forget, because you’re already working with other people by that time.” [P14]*

The patient’s perspective created better understanding of the responses and actions of patients during delirious episodes. Participants often had the notion that delirious patients were acting weird, but after seeing the patient’s perspective in ‘The Delirium Experience’ they were able to make more sense of the responses. Furthermore, a participant explained that, after playing the game, she was able to understand why patients do not respond when you enter their room: *“Something I also didn’t realize before, was that when he wakes up with all the little creatures crawling around, the nurse enters the room. But even I almost didn’t notice her, because you’re so focused on what’s happening around you.” [P15]*

Participants also mentioned that the patient’s perspective showed them important aspects of providing care to delirious patients. The game made them experience the effects of their actions on the patient. A participant explained: *“I think the patient’s perspective is really important to realize why it’s frustrating not to be able to talk to somebody. You can read that you should first communicate with the patient … but when you hear doctors speak from his perspective, you realize that you also don’t like people speaking across you while you can’t hear them. That reinforces the sense of why you should do something and probably you’ll remember that experience, because you haven’t only read it, but also experienced it”. [P14]*

Experiencing the patient’s perspective also made participants reflect on their actions as a healthcare professional. Participants mentioned that they will act differently when they encounter delirious patients, which was explained as follows: *I’ve learned a lot, and that’s why I’ll pay more attention to a delirious patient. It is something really intense, and something small can already have a big impact on a patient. So, now I’ve seen this, I think I’ll take delirium more seriously.” [P7]*

Before playing The Delirium Experience, participants stated that they sometimes felt helpless when caring for delirious patients, because they felt they were not able to do enough. After playing the game, participants mentioned that delirious episodes were much more intense than they had expected.

#### Nurse

The nurse’s perspective mainly provided the participants with examples of how to provide good quality care to delirious patients. This made them more aware of both which behaviors support or hinder delirium care delivery and how to communicate with patients suffering from delirium. A participant explained what she learned from the nurse’s perspective: *“Anyway, I learned how I should approach [and behave to] these patients.” [P2]* It also made participants who were insecure or unsure, who had the feeling that they were not able to do enough for delirious patients, feel less helpless. Furthermore, participants stated that the nurse’s perspective was important for improving collaboration between nurses and doctors in delirium care. The nurse’s perspective allowed participants to gain more insight into the daily routines and tasks of a nurse which, in turn, could improve their understanding and foster future collaborations between nurses and physicians.

### Interactivity to experiment

In the serious game, players have different options to choose from, thereby allowing them to experiment with care actions. We refer to this as interactivity in the game. The interactive element of the narrative allows the participants to change its course. Participants stated that, because of the interactivity in ‘The Delirium Experience’, they had obtained knowledge on how to provide delirium care. This was mainly because the interactivity engaged them and actively involved them in the study material, which made them reflect on their actions in the game and the consequences of these actions. Participants mentioned that this also made them reflect on how to provide delirium care in real life. One participant answered: “*It looked realistic and in this way you engage in caring for delirious patients without doing something wrong to [i.e. fear of harming] a real patient. The choices [offered in the game] are, of course, somewhat limited, in a way that doesn’t resemble how you’d act in real life, but it makes you think about what you should do.” [P9]* Participants’ reflection on what they would do in real life was even enhanced by the interactivity, because real patients’ wellbeing may also depend on their choices. Furthermore, the interactivity made the participants more involved, because it gave them a feeling of control over the course of the narrative. Participants valued the possibility to make choices within ‘The Delirium Experience’, because it allowed them to make mistakes and learn from these mistakes without harming a patient. Finally, the interactivity made the participants curious to explore other options and made them want to play the game multiple times.

### Realism

The Delirium Experience is a video-game that uses real actors and, therefore, it has high levels of realism. Participants experienced the game as realistic and felt this was important for them for several reasons. First, playing the game created a better understanding of both how patients experience delirious episodes and how the episodes influence patients’ behaviors. Their understanding was further enhanced by listening to the audible thoughts of the patient during the delirious episodes, which were included in the game.

Second, the realism in ‘The Delirium Experience’ made it easier for them to transfer what they had learned to real practice. (a) It became more easy to relate the examples of how to provide delirium care or communicate with a delirious patient to real life scenario’s. (b) The limited amount of time that was available to provide care to the patient resembled real practice. This made participants thoughts focus on what would be the best care choice to achieve the best possible outcome for the patient’s wellbeing. A participant explained: *“Because of the limited amount of time available you can’t do everything you want … Because of that you start thinking about what would be the most important thing you could do for a delirious patient … But if I could do everything [try every option], I would have clicked on everything and just see what would happen. [P14]*

Third, participants felt more involved in the study material, because it looked realistic to them. The realistic responses of the patient interested them and the game’s realism encouraged them to make more deliberate choices, as explained by a participant: *“I like it [the realism], because you get more involved in the narrative, and it’s not just an icon, but a real person … that’s nice because it creates more empathy and, because of that, you make more deliberate choices instead of just clicking on something.” [P3]*

### Feedback

In The Delirium Experience, players receive written feedback as well as feedback that is incorporated in the narrative. In general, the participants stated that the feedback in the game gave them more knowledge about actions that should be taken when providing delirium care. In particular, the written feedback at the end of each day in the game was perceived to improve delirium care knowledge. However, participants also indicated a need for more specific written feedback, providing information that explains why certain choices are incorrect and how to improve even more. Yet, the lack of this kind of feedback also triggered them to experiment with different options and made them reflect on their choices, as one participant answered: “*At the end, I didn’t know what I did wrong. I think it was the order of actions, but what the correct order was, I just don’t know. … this didn’t work, so I’ll try something else, or just think about what would be the correct order. But I missed to see the correct choices, because you can learn from that. But thanks to that, I started thinking about the choices I made, like ‘..then this happened, what would have happened if I’d done this, would that have been the correct choice or should I … ’ So actually, it made me think about the choices.” [P6]*

In contrast, other participants mentioned that the patient’s responses and the progression of the delirious episodes had triggered them to reflect on their actions and how these had influenced both the patient and the narrative. They used the feedback derived from the patient’s responses to actively search for answers on why something was wrong or how to improve themselves, as explained by a participant: “*So I think the way the patient reacts and how his story continues, that’s more important. Because of that, you see what effect your choices have on the patient himself. And then you can think of what you should or shouldn’t do.” [P13]*

## Discussion

In this study, we aimed to explore aspects that could potentially affect students’ attitudes and learning experiences regarding delirium by making use of a serious games’ narrative. We identified four important aspects at play when learning how to provide care to delirious patients: (1) perspectives of the people involved in delirium care, (2) interactivity to experiment, (3) realistic views and care options, and (4) feedback on care actions.

### Principal findings

#### Attitude

We found several aspects of the serious game’s narrative that played a role in addressing students’ attitudes towards delirium in terms of affective, behavioral, and cognitive components of attitude. The affective component of attitude resonated with feelings and emotions towards delirium [44, 45]. Aspects from The Delirium Experience that influenced students’ feelings and emotions were: the patient’s perspective, the nurse’s perspective, and the realism of the delirious episodes. These aspects made students feel less helpless when it comes to providing care to a delirious patient and helped them gain a better understanding of what delirious patients encounter and how this can explain their behaviors. We consider this an important finding since a good understanding of how frightening delirium can be, is often lacking [4]. Healthcare professionals often experience discomfort when caring for delirious patients [17, 19].

The behavioral component of attitude refers to a person’s tendency to behave in a certain way towards delirium [44, 45]. The patient’s perspective, the nurse’s perspective and interactivity of the narrative appeared to influence the behavioral component. These three aspects contributed to students’ awareness of how to behave towards delirious patients. The patient’s perspective made them aware of how patients experience healthcare professionals’ behaviors; the nurse’s perspective showed them how to execute these behaviors; and the interactivity supported them in seeing the consequences of their behaviors. As a consequence, students often indicated that communication with a delirious patient was a behavior they would like to change. This is one of the important topics that need to be addressed in delirium education [4].

The cognitive attitudinal component consists of students’ knowledge and beliefs on delirium [44, 45]. Aspects of the narrative of ‘The Delirium Experience’ that affected the cognitive component were: the patient’s perspective, interactivity and feedback. They changed students’ beliefs of how their actions as a healthcare professionals could make a difference for patients experiencing delirious episodes and, therefore, hold the potential to change students’ attitudes [59]. Additionally, students felt that these aspects had increased their knowledge of how patients experience delirium and how to provide delirium care.

The patient’s perspective played an important role in fostering all three attitudinal components. Therefore, we emphasize the need to incorporate patient experiences and perspectives into delirium education. The results of our study highlight the importance of a patient-oriented approach to delirium education in order to drive attitudinal change [4, 6, 7].

Aspects that were found to affect students’ attitudes towards delirium were also found to encourage involvement in ‘The Delirium Experience’. Previous research in domains outside medical education already showed that the use of multiple perspectives, interactivity, and realistic settings can enhance players’ involvement [41, 60, 61]. To bring about attitudinal change, it is important to ensure a high level of involvement, because it motivates students to evaluate and reflect upon the messages incorporated in the game’s narrative. These evaluations and reflections are related to attitudes that are more persistent over time and may be a better predictor of students’ behavior in providing delirium care [62, 63].

#### Learning experiences

Multiple perspectives, interactivity, and realism were also found to foster students’ learning experiences. These aspects encouraged active involvement in the learning material and helped students realize the value of what they could learn from the game’s narrative. This is an important finding since, in delirium education, there is a growing demand for interactive methods that actively involve learners [3, 5, 14, 20]. Active involvement improves both learning experiences and learning outcomes [64, 65]. Our results suggest that if we want to enhance active involvement in delirium education, it is important to include the perspectives of people involved in delirium care in the learning material and to stay close to reality.

Many aspects of the game’s narratives triggered affective responses in students, for example, the frightening delirious episodes from the patient’s perspective and students’ unexpected influence on delirious episodes due to the interactivity. Affective responses are important to enhance students’ learning experiences and understanding of the patient’s experience [48, 50, 66, 67]. In delirium care, however, healthcare professionals’ understanding of the patient’s experience is often lacking [3]. Therefore, affective responses are of essential value in delirium education. In addition, the patient’s perspective contributes to a patient-oriented approach to education, which has been presented as an important improvement for delirium education [3, 6, 22]. Our findings suggest that the patient’s perspective evokes affective responses in students, which may eventually contribute to better delirium care by facilitating the transfer of knowledge to practical performance [47].

Incorporating different forms of feedback in a narrative seems to provoke active experimentation and reflection, which is important for learning [48]. Feedback is most effective when the messages are specific and clear [68, 69]. The results of our study suggest that students may use a different strategy to achieve the learning goals, when the provided feedback is not specific enough for them. The general, written feedback on students’ progression triggered some students to actively experiment with the narrative and search for the feedback they needed. In delirium care, active experimentation plays an important role in healthcare professionals’ learning [14, 48]. Students also reflected on their care actions to find answers to unresolved questions that had not been addressed in the written feedback. Such reflections may foster transfer of obtained knowledge into practice, because developing and participating in professional practice requires constant reflection on action and exploration of multiple perspectives [49]. However, students who had not been triggered to search for explanations in an alternative way felt that the written feedback they had received was insufficient to meet their learning needs. It seems that these students were unable to decode the feedback incorporated in the patient’s narrative [70].

### Strengths and limitations

An important strength of our study is that it responds to the call for more insight into aspects that contribute to achieving goals in both delirium education and serious games [1, 3, 4, 25–27, 71, 72]. Our findings can be used to inform future development of delirium education and serious games.

Our study has several of limitations. First, the aim of our study was to investigate aspects of a serious game’s narrative that are at play to improve delirium education. Although we had explained this to our participants, we noticed that it was hard for them to distinguish between aspects of the narrative and aspects of the serious game. Consequently, our findings may not be generalizable to narratives in other mediums.

Second, our study aimed to explore students’ perceptions, so we did not objectively observe or evaluate actual improvement. Consequently, the aspects we identified hold the potential to affect students’ attitudes and learning experiences, as described in this study. Third, since we used the complete version of The Delirium Experience, we were not able to isolate specific aspects and investigate their individual effects. The aspects we identified were always presented to the participants in combination with other aspects of ‘The Delirium Experience’. However, this study provides insight into aspects that are important for both delirium education and serious games, which may inform future design or, in the developmental phase, help distinguish between aspects [73].

### Recommendations for further research

Interprofessional cooperation is often not addressed in current delirium education [1, 18]. Our results suggest, that understanding another person’s perspective can enhance co-operation between healthcare professionals. It will be more easy for medical students to collaborate with nurses, if they have more insight into the nurses’ activities. Therefore, we propose to investigate the use of perspective-taking to improve interprofessional cooperation in delirium care.

In this study, we were interested in the use of a narrative as a learning tool for undergraduate students, especially, since researchers emphasized the need to teach delirium care at an early stage in education [6]. However, similar issues need to be addressed to educate graduated healthcare professionals who are already working with delirious patients. Therefore, we recommend to replicate this study using a broader sample of healthcare professionals.

As explained earlier, some players actively experimented with the feedback incorporated into the game’s narrative to find answers to their questions. However, there were also players who had not been triggered to find answers to their questions in this way. They valued the game’s feedback as insufficient. Further research is needed to understand how we can guide these students in learning how to use other types of feedback to fulfill their learning needs, especially since research on the impact of learner characteristics on the effectiveness of feedback in serious games is sparse [74]. A strategy that may support students in making use of different feedback types is modeling, which involves showing how to solve a particular problem or which information is important to solve this problem [32]. Serious games that make use of modeling support players in selecting relevant information and improve the learning potential [32]. With regard to The Delirium Experience, we think that it is important to embed the game in the curriculum together with face-to-face plenary debriefings to fulfill students’ feedback needs [75, 76].

Our findings suggest that, in case of delirium education, realistic design of options and settings can improve reflection and, consequently, promote knowledge integration. This contradicts earlier reviews showing that schematic, textual or cartoonlike visual designs are often equally or more effective than realistic serious games [32, 77]. However, it was also suggested that further research is needed on how levels of realism can enhance learning outcomes in specific domains [77]. We argue that, in case of delirium or other patient-oriented education, a realistic design can support learners in interpreting and making sense of the experience, but this needs to be further studied.

## Conclusion

In a serious game to support delirium care education, several aspects of the game’s narrative can improve students’ learning experiences: the perspectives of people involved in the care process, interactivity to experiment, realistic views and care options, and feedback on care actions. This creates greater understanding of a delirious patient’s experience and addresses attitudes towards delirious patients, but also contributes to knowledge on care behaviors and how to communicate with delirious patients. A patient-oriented narrative within a serious game provides students with an experience in which reflection plays an important role. It is also a medium for active experimentation to promote a better understanding of how healthcare professionals can influence the patient’s experience of delirium.

## Supplementary information

**Additional file 1.** Interview guide.

## Data Availability

The transcripts of the interviews are available from the corresponding author on reasonable request.

## Abbreviations

ICMJE: International Committee of Medical Journal Editors
KBS: Kiki Buijs-Spanjers
AH: Anne Harmsen
HH: Harianne Hegge
JS: Jorinde Spook
SR: Sophia de Rooij
DJ: Debbie Jaarsma
