# Supplementary material for: The influence of a serious game’s narrative on students’ attitudes and learning experiences regarding delirium: an interview study
Source: BMC Med Educ. 2020 Sep 1;20:289. doi: 10.1186/s12909-020-02210-5 (PMC7465326; doi:10.1186/s12909-020-02210-5)
Supplement: Supplementary file 1 — Additional file 1. Interview guide. [file 12909_2020_2210_MOESM1_ESM.docx]

# Additional file 1 - Interview guide

- Introduce
- Aim and time investment
- Informed consent
- Background information: gender, age, education, experience, experience with delirium

| **1** | **2** | **3** | **Probes** |
| --- | --- | --- | --- |
| Can you summarize the narrative of The Delirium Experience? | | | |
|  | | | |
| What did the narrative of The Delirium Experience mean to you? | | | |
|  | What did the narrative mean for how you think about delirious patients? | | - Storyline - Engagement/involvement - Realism - Characters - Identification/recognition - Responsibility - Interactivity |
|  | | What do you know about delirious patients? |  |
|  |  | What do you think of delirious patients?  How do you see delirious patients?  How did the narrative influence your feelings towards delirious patients? |  |
|  |  | How to you behave towards someone with delirium?  How do you care for someone with delirium?  How did the narrative influence how you would behave to someone with delirium? |  |
|  | What did the narrative mean for your learning experience? | |  |
|  | | What did you learn about delirious patients because of the narrative?  Which new knowledge did you gain because of the narrative? |  |
|  | | What is your experience with learning in this way?  Which aspects of the narrative contribute to this experience? |  |
|  |  | How did the narrative change your knowledge on delirious patients?  Which aspects of the narrative contribute to this change? |  |
|  |  | How did the narrative make you reflect upon your knowledge / behavior / actions?  Which aspects of the narrative contribute to this reflection? |  |
|  |  | How did you experience the feedback in the narrative?  What kinds of feedback did you recognize?  What did the feedback mean to your learning experience? |  |
|  |  | Why will/won’t you apply your new knowledge into practice? |  |
| Is there anything else you would like to share or add? | | | |
